# Amyloid Beta Peptide Slows Down Sensory-Induced Hippocampal Oscillations

**DOI:** 10.1155/2012/236289

**Published:** 2012-04-29

**Authors:** Fernando Peña-Ortega, Ramón Bernal-Pedraza

**Affiliations:** ^1^Departamento de Neurobiología del Desarrollo y Neurofisiología, Instituto de Neurobiología, Universidad Nacional Autónoma de México, UNAM-Campus Juriquilla, 76230 Juriquilla, QRO, Mexico; ^2^Departamento de Farmacobiología, Cinvestav-IPN, Mexico City, DF, Mexico

## Abstract

Alzheimer's disease (AD) progresses with a deterioration of hippocampal function that is likely induced by amyloid beta (A**β**) oligomers. Hippocampal function is strongly dependent on theta rhythm, and disruptions in this rhythm have been related to the reduction of cognitive performance in AD. Accordingly, both AD patients and AD-transgenic mice show an increase in theta rhythm at rest but a reduction in cognitive-induced theta rhythm. We have previously found that monomers of the short sequence of A**β** (peptide 25–35) reduce sensory-induced theta oscillations. However, considering on the one hand that different A**β** sequences differentially affect hippocampal oscillations and on the other hand that A**β** oligomers seem to be responsible for the cognitive decline observed in AD, here we aimed to explore the effect of A**β** oligomers on sensory-induced theta rhythm. Our results show that intracisternal injection of A**β**1–42 oligomers, which has no significant effect on spontaneous hippocampal activity, disrupts the induction of theta rhythm upon sensory stimulation. Instead of increasing the power in the theta band, the hippocampus of A**β**-treated animals responds to sensory stimulation (tail pinch) with an increase in lower frequencies. These findings demonstrate that A**β** alters induced theta rhythm, providing an *in vivo* model to test for therapeutic approaches to overcome A**β**-induced hippocampal and cognitive dysfunctions.

## 1. Introduction

Alzheimer's disease (AD), the most common form of dementia, is characterized by a progressive decline in cognitive function [1–5] that correlates with the extracellular accumulation of amyloid beta protein (A*β*) [1, 4, 5]. Deterioration of hippocampal function, likely induced by A*β* oligomers, contributes to the memory deficits associated with Alzheimer's disease (AD) [5–8]. Normal hippocampal function is strongly dependent on a 3 to 10 Hz oscillatory activity, namely, the theta rhythm [9–11]. Theta oscillations have been associated with various cognitive processes in several species, including humans [9–11]. Theta rhythm abnormalities are usually related to memory deficits and pathological changes in the brain [12–14]. In fact, subjects with AD show a typical “electroencephalographic slowing” that includes increased slow rhythms and decreased fast rhythms [6, 13, 15, 16]. Regarding theta rhythm, AD patients show increased theta rhythm at rest [6, 15, 16], but they also show a decrease in induced-theta rhythm; both of these changes in theta rhythm correlate with a reduced cognitive performance [17]. A similar contradictory scenario has been found in transgenic mice that overproduce A*β* and exhibit AD-like symptoms [18, 19]. The complex relationships between AD pathology and theta rhythms have been explained by the theta rhythm heterogeneity that exists both in humans and in mice [12, 20]. Experimentally, the reduction in resting hippocampal theta rhythm has been mimicked by A*β* application, both *in vitro* [21–23] and *in vivo* [24, 25]. However, just one previous study has shown that intracerebroventricular injection of monomers of a short A*β* sequence (peptide 25–35) decreases the power of the induced theta rhythm [26]. This finding still needs to be confirmed because different A*β* peptides, as well as their aggregation states, differentially affect similar hippocampal rhythms [27]. Thus, in this study we explored the effect of oligomers of the full-length A*β* sequence (peptide 1–42) on induced theta rhythm *in vivo*. The use of A*β*1–42 oligomers has more relevance for the study of AD-related neural network disruption since early symptoms of AD are better correlated with the amount of soluble A*β* than other histopathological makers [2, 3]. Our data show that intracisternal application of A*β* slows down sensory-induced hippocampal oscillations, supplanting theta oscillations with a slower rhythm.

## 2. Materials and Methods

Experimental protocols were approved by The Local Committees of Ethics on Animal Experimentation (CICUAL-Cinvestav and INB-UNAM) and followed the regulations established in the Mexican Official Norm for the Use and Care of Laboratory Animals (“Norma Oficial Mexicana” NOM-062-ZOO-1999). For these experiments, Wistar rats (300–330 g) were briefly and lightly anesthetized with ether vapor just before receiving a single, intracisternal injection of 5 *μ*L of either vehicle (F12 medium) or oligomerized A*β*1–42 (5 and 50 pmoles). The injector was connected to a Hamilton syringe mounted on dual perfusion pump (Harvard Apparatus Co., MA, USA). Animals were allowed to recover for 1 h after the intracisternal injection. Then, the animals were anesthetized with urethane (1.3 g/Kg; i.p.) and secured in a Kopf stereotaxic frame with the nose bar positioned at −3.3 mm [28, 29]. A bipolar electrode was implanted in the left dorsal hippocampus (*A* = −3.6 mm *L* = 2.4 mm and *V* = 4.2 mm from bregma, according to the atlas of Paxinos and Watson [30]) using standard stereotaxic procedures. The electrodes were attached to male connector pins, which were inserted into a connector strip. Hippocampal field recordings were amplified and filtered (highpass, 0.5 Hz; lowpass, 1.5 KHz) with a wideband AC amplifier (Grass Instruments, Quincy, MA, USA). Theta rhythm was elicited with sensory stimulation, consisting of a tail pinch produced by a plastic clamp positioned on the tail 2 cm from its base. A tail pinch, lasting 75 s, was applied each 10–20 min for at least 1 h. At the end of the hippocampal field recordings, all animals were processed for histological location of the electrode [28, 29, 31]. The recording site was visually confirmed to be located in the hippocampal fissure.

All recordings were digitized at 3–9 KHz and stored on a personal computer with an acquisition system from National Instruments (Austin, TX, USA) by using custom-made software designed in the LabView environment. The recordings obtained were analyzed offline by performing classical power spectrum analysis with a resolution of 0.61 Hz [26, 27, 32]. Segments of 30 sec were analyzed using a Rapid Fourier Transform Algorithm, with a Hamming window, in Clampfit (Molecular Devices). The power spectra during the tail pinch, at any given frequency, were also divided by their corresponding prestimulus power spectra and expressed as percentage of control (100% meaning no difference between tail-pinch and prestimulus power spectra). The mean difference spectra were then calculated by averaging the differences obtained in any given group [33–35]. For time-frequency analysis, segments of 40 s were analyzed using the Morlet wavelet basis and plotted as a time-frequency representation (TFR) [26, 32]. 

Data are expressed as mean ± standard error of mean (SEM). To analyze the data, the Wilcoxon signed-rank test was used to compare control versus tail-pinch spectra in the same group of animals. The Mann-Whitney *U* test was used to compare groups. A value of *P* < 0.05 was accepted as significant.

## 3. Results

**Figure 1:**
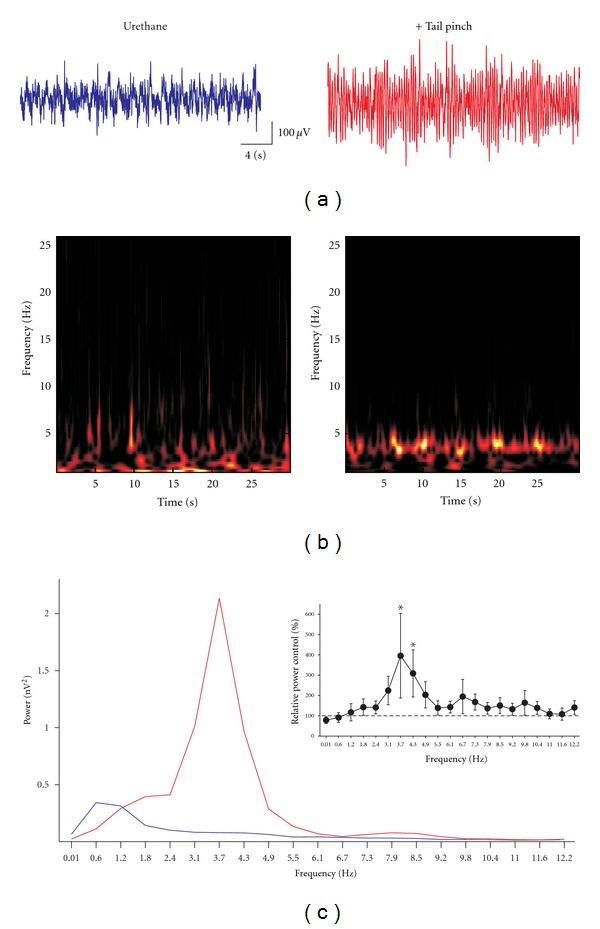
Sensory stimulation induces hippocampal theta oscillations. (a) Representative field recordings obtained from the hippocampal fissure in a urethane-anaesthetized rat at rest (blue recording) and upon sensory stimulation (red trace). (b) and (c) show the spectrograms and the power spectra, respectively, of the traces shown in (a). The blue power spectrum corresponds to the recording at rest, and the red power spectrum corresponds to the recording upon sensory stimulation. The inset in (c) shows the quantification of the change in power upon sensory stimulation, compared with basal hippocampal activity. *Indicates a significant difference compared to the control (*P* < 0.05; Wilcoxon signed-rank test).

Under urethane anesthesia, hippocampal local field potential showed a pattern of irregular activity (Figure 1(a); blue trace) that resembles the so-called large amplitude irregular activity (LIA) and that corresponds to the activity observed during immobility and slow-wave sleep [9, 12]. Such activity turns into more steady, oscillatory activity upon sensory stimulation (tail pinch; Figure 1(a); red trace). The spectrograms show that basal hippocampal activity under urethane anesthesia consists of a variable mixture of frequency components that vary over time (Figure 1(b)). In contrast, upon sensory stimulation, hippocampal activity exhibits a more constant oscillatory pattern (Figure 1(b)). The power spectrum shows that basal hippocampal activity under urethane anesthesia peaks at 2.5 ± 0.5 Hz, whereas theta rhythm has a frequency of 3.0 ± 0.4 Hz (Figure 1(b)). Quantification of the change in power upon tail pinch, compared with basal hippocampal activity, shows that sensory stimulation significantly increases the power in the low theta range (3.7–4.3 Hz) (Figure 1(c); inset).

**Figure 2:**
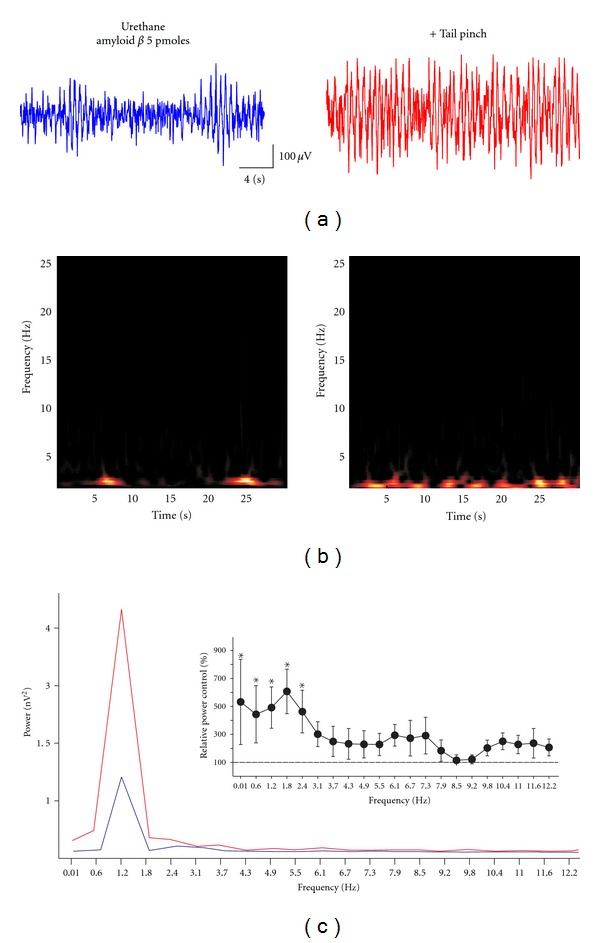
Effect of 5 pmoles amyloid beta on the sensory-induced hippocampal theta oscillations. (a) Representative field recordings obtained from the hippocampal fissure in a urethane-anaesthetized rat at rest (blue recording) and upon sensory stimulation (red recording). (b) and (c) show the spectrograms and the power spectra, respectively, of the traces shown in (a). The blue spectrum corresponds to the recording at rest, and the red power spectrum corresponds to the recording upon sensory stimulation. The inset in (c) shows the quantification of change in power upon sensory stimulation, compared with basal hippocampal activity. *Indicates a significant difference compared to control (*P* < 0.05; Wilcoxon signed-rank test).

**Figure 3:**
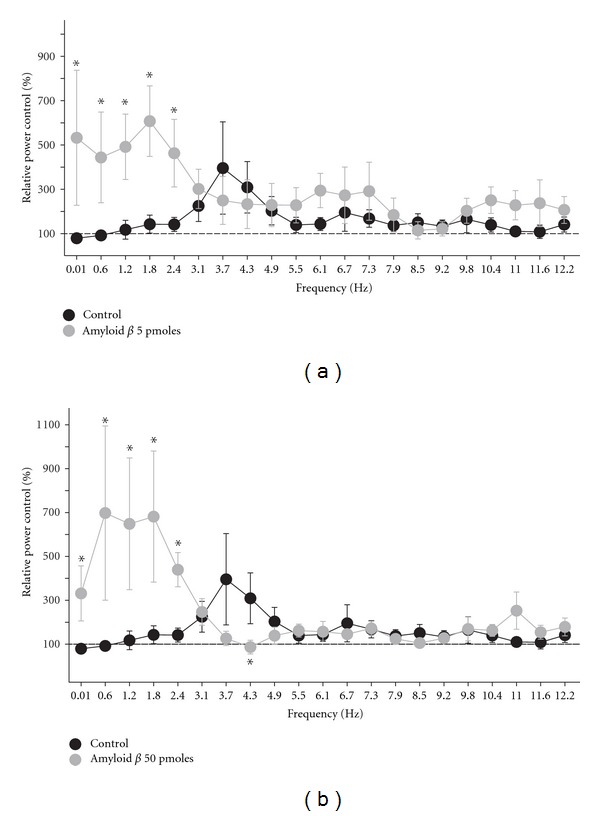
Amyloid beta slows, in a dose-dependent manner, the oscillatory activity induced by sensory stimulation. Change in power induced by sensory stimulation in control rats (black circles; *n* = 9) compared to that in amyloid beta-injected rats (gray circles; *n* = 6). Animals were injected with two doses of amyloid beta. With 5 pmoles (a), the increase in power, upon sensory stimulation, shifts towards slow frequencies. Injection of 50 pmoles of amyloid beta (b) also shifts the increase in power, upon sensory stimulation, towards slow frequencies, and it also significantly reduces the increase in theta rhythm. *Indicates a significant difference compared to control rats (*P* < 0.05; Mann-Whitney *U* test).

When testing the effects of A*β* oligomers on hippocampal activity, we did not find any significant difference in the hippocampal activity compared with control animals, due to the high variability among groups, either in power or peak frequency, due to the high variability among groups (Table 1). As illustrated in Figure 2, the hippocampal activity recorded after intracisternal injection of 5 pmoles of A*β* oligomers is still characterized by a pattern of nonstationary, irregular activity under urethane anesthesia (Figure 2(a); blue trace). This activity also turns into a more homogeneous oscillatory activity upon sensory stimulation (tail pinch; Figures 2(a), 2(b); red trace). The spectrograms show that basal hippocampal activity under urethane anesthesia consists of a variable mixture of frequency components that change over time (Figure 2(b)). In contrast, upon sensory stimulation hippocampal activity turns into a more stationary, oscillatory state (Figure 2(b)). On average, in animals injected with 5 pmoles of A*β* oligomers and under urethane anesthesia, basal hippocampal activity peaks at 3.4 ± 0.6 Hz, and the tail pinch-induced rhythm has a frequency of 3.4 ± 0.6 Hz (Table 1). As mentioned, neither the power nor the peak frequency of hippocampal activity changed upon A*β* application in either basal or sensory-stimulated conditions (Table 1). However, quantification of the change in power upon tail pinch shows significant changes compared with basal hippocampal activity. Sensory stimulation in A*β*-treated animals significantly increases the power in low frequencies (0.01–2.4 Hz) (Figure 2(c); inset and Figure 3). In fact, the increase in power of those frequencies was significantly higher in A*β*-treated animals than in control (vehicle-treated) animals (Figure 3). Although sensory-induced theta rhythm was not significantly changed relative to control animals by injection of 5 pmoles of A*β*, it was significantly reduced at 4.3 Hz by a higher dose, 50 pmoles of A*β* (Figure 3).

## 4. Discussion

Our results show that intracisternal application of A*β*1–42 oligomers does not produce any significant effect on spontaneous hippocampal activity, but it disrupts the hippocampal activation induced by sensory stimulation. A*β*-treated animals do respond to sensory stimulation (tail pinch), but the increase occurs in lower frequencies than in control animals. These findings may correlate with the EEG slowing observed in AD patients [6, 13, 15, 16] as well as with the reduction in evoked theta rhythm [17] that was also observed in AD patients. In our previous report, we demonstrated that intracerebroventricular injection of monomers of the short A*β* sequence (25–35) reduced the power of induced theta rhythm [26]. However, in that study we did not find the change in theta frequency observed here. The simplest explanation for this difference is that oligomers of A*β*1–42 may act on different cellular targets and produce different effects than monomers of A*β*25–35 [27]. If so, without ignoring the advantages of using monomers of A*β*25–35 [23, 26], we believe that the use of A*β*1–42 oligomers may represent a more valid model to explore some of the changes related to AD pathology. A second potential explanation is that in the current study we used intracisternal application of A*β* in contrast to the intracerebroventricular injections used previously [26]. It has been found that intracerebroventricular and intracisternal administration of the same substance do not always produce the same effect, probably due to differences in the brain structures preferentially reached by the injection in those sites, as well as to the different concentrations of the injected substance reached at those structures [36–42].

Our results are in agreement with previous findings that direct application of A*β*, either in the medial septum or in the hippocampus, reduces theta-rhythm power both *in vivo* and *in vitro* [22–26, 43]. However, in our hands, intracisternal application of A*β* also shifts the frequency of sensory-evoked oscillations to the left. Several factors have been associated with the reduction in theta power. For instance, we have shown that this reduction is related to a reduction in intrahippocampal glutamatergic transmission [22, 26], but the reduction in power also has been associated with the blockade of several K+ channels [23, 44] or with A*β*-induced changes in septal neuron firing [23–25]. The shift in frequency induced by A*β* might be related to changes in the activity of interneurons in the hippocampus or elsewhere [23–25] or to the effect of A*β* on transient potassium currents [44]. Overall, the effects of A*β* on hippocampal theta rhythm seem to involve a complex mixture of effects on several neural types within several neural networks. It is well known that hippocampal theta rhythm could be affected by a decoupling of one or several autonomous oscillators within the hippocampus [45] or in other interconnected neural networks [24, 25]. Correlative *in vitro* experiments are required to corroborate this hypothesis and to determine viable molecular targets to prevent A*β*-induced neural network disruption.

## Figures and Tables

**Table 1:** Power and peak frequency of the hippocampal activity recorded in anaesthetized animals in control conditions and after the intracisternal injection of amyloid beta (A*β*). No significant differences were observed among or within groups.

Condition	Power (nV^2^)	Peak Frequency (Hz)
Urethane	4.3 ± 2.5	2.5 ± 0.5
+ Tail pinch	5.1 ± 2.6	3.0 ± 0.4
Urethane + A*β* 5 pmoles	1.2 ± 1.0	3.4 ± 0.6
+ Tail pinch	1.5 ± 1.3	3.4 ± 0.6
Urethane + A*β* 50 pmoles	2.9 ± 1.7	3.9 ± 0.1
+ Tail pinch	3.6 ± 2.1	3.4 ± 0.4
